# Mechanistic insights into hydroxynaphthoic acid-based suppression of lignin repolymerization

**DOI:** 10.1039/d5su00841g

**Published:** 2026-04-13

**Authors:** Chenhao Li, Matthias Alexander Ulrich Eckl, Pascal Fitz, Thomas Pielhop, Sergio Vernuccio

**Affiliations:** a School of Chemical, Materials and Biological Engineering, The University of Sheffield Sheffield UK; b School of Chemistry and Chemical Engineering, University of Southampton Southampton UK s.vernuccio@soton.ac.uk; c Institute of Chemistry and Biotechnology, Zurich University of Applied Sciences Zurich Switzerland thomas.pielhop@zhaw.ch

## Abstract

As a renewable biomass resource, lignocellulose holds significant potential for the production of chemicals and fuels, due to its vast availability and low cost. However, its complex and chemically stable structure poses a major challenge to its efficient utilization. Acidic pretreatment methods (including autohydrolysis) are widely employed to improve the conversion efficiency of lignocellulose into valuable chemicals. While the acidic environment promotes the breakdown of the lignocellulose structure, facilitating its (bio)chemical conversion, it also induces lignin repolymerization reactions, thereby hindering the pretreatment and further valorisation of cellulose and lignin. The use of carbocation scavengers to cap reactive sites (carbocations) formed during the pretreatment process in lignin, has recently proven to be an effective method for mitigating these issues. Therefore, the discovery and evaluation of effective scavengers has emerged as a key research focus in lignocellulose valorisation. In this study, we investigate the performance of three hydroxynaphthoic acid isomers – 3-hydroxy-2-naphthoic acid, 6-hydroxy-1-naphthoic acid, and 6-hydroxy-2-naphthoic acid – as potential scavengers for suppressing lignin repolymerization. Fukui function calculations were employed to determine the most favourable nucleophilic sites within each compound. In addition, the acid-catalysed dehydration and cation scavenging processes were simulated using a representative lignin cluster model. Our results demonstrate that the binding interactions between the scavengers and the lignin-derived carbocations at favorable active sites play a critical role in suppressing repolymerization. Among the hydroxynaphthoic acid tested, 3-hydroxy-2-naphthoic acid exhibited the strongest binding affinity with the lignin carbocation (−16.8 kJ mol^−1^) and the most significant scavenging effect. This result was further validated experimentally, by studying the consumption of the three hydroxynaphthoic acids and their effect on the lignocellulose structure in the autohydrolysis pretreatment of spruce wood. Among the investigated scavengers, 3-hydroxy-2-naphthoic acid showed the lowest recovery (2%) after pretreatment at 210 °C, indicating its high reactivity. Moreover, evaluation of the scavenger effect on lignocellulose enzymatic digestibility revealed that 3-hydroxy-2-naphthoic resulted in the highest glucose yield.

## Abstract 2

Acidic pretreatment of lignocellulosic biomass facilitates its conversion into valuable chemicals and fuels. The use of carbocation scavengers to cap lignin reactive sites helps mitigate undesired repolymerization reactions.

## Graphical abstract

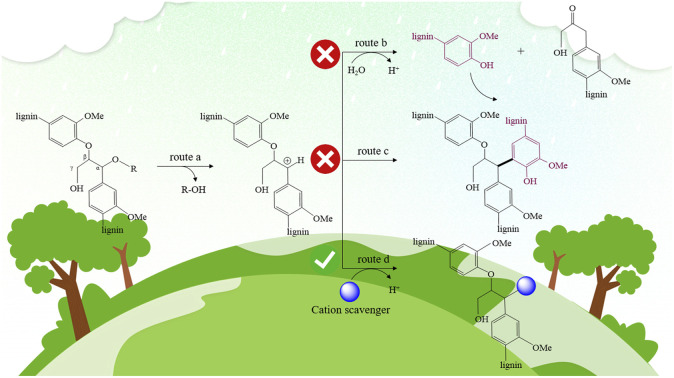

**Sustainability spotlight**

Efficient utilization of lignocellulosic biomass is a cornerstone of sustainable chemical production, yet the repolymerization of lignin during acidic pretreatment remains a major obstacle to its efficient utilization. This study advances sustainability by further developing a novel method for the conversion of lignocellulosic biomass. The approach involves the use of cation scavengers for suppressing undesired lignin repolymerisation in biomass pretreatment. This strategy enhances cellulose bioconversion, offering a sustainable alternative to non-renewable fossil resources. This research primarily aligns with UN Sustainable Development Goal 7: Affordable and Clean Energy, and SDG 12: Responsible Consumption and Production, by promoting renewable biomass as a sustainable resource for fuels and chemicals. It also supports SDG 13: Climate Action, through its contribution to carbon-neutral production pathways.

## 1 Introduction

The extensive use of fossil resources and its contribution to global warming have become major obstacles to the sustainable development of the global economy and society, demanding urgent and effective solutions. Using renewable biomass resources, especially lignocellulose materials, to produce liquid fuels and essential bulk chemicals offers a promising approach to address these issues.^1^ This strategy offers a sustainable alternative to the diminishing reserves of non-renewable fossil resources, such as petroleum, thereby contributing to long-term societal and economic progress.^2,3^

**Fig. 1:**
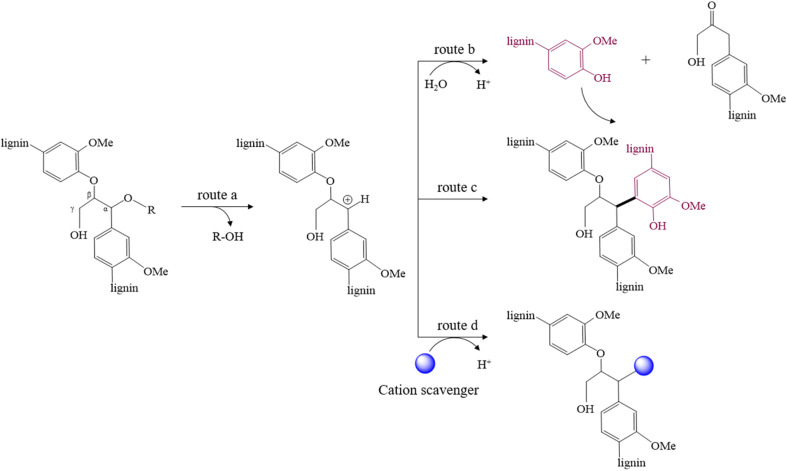
Reaction of lignin in acidic media: (a) acid catalysed depolymerization and formation of carbocation, (b) cleavage of β-O-4′ bond and depolymerization, (c) lignin repolymerization reaction, (d) scavenging of carbocation. Adapted from Pielhop, 2023.^24^

Lignocellulose has a complex structure, primarily consisting of three major components: cellulose, hemicellulose and lignin. These constituents coexist within the plant cell wall in various configurations, where they are closely intertwined and connected. Structurally, cellulose forms a rigid framework that is embedded with hemicellulose and lignin complexes, resulting in a heterogeneous architecture. These components are interconnected through a variety of chemical bonds, which contribute to the inherent recalcitrance of lignocellulose.^4,5^ As a result, the efficiency of direct bioconversion of lignocellulose is typically low, necessitating pretreatment to enhance the conversion process.^5,6^ Among various pretreatment strategies, autohydrolysis, acid pretreatment, or lignin-removing organosolv processes have proven effective for facilitating the subsequent enzymatic or microbial processing.^7^ The addition of acid catalysts or the *in situ* release of acids from hemicellulose during the pretreatment (autohydrolysis) facilitates the depolymerisation of lignocellulosic biomass and promotes the formation of lignin-derived carbocations (denoted as lignin^+^) (Fig. 1, route a).^8^ Subsequent depolymerisation cleaves the β-arylether linkages of lignin^+^, resulting in the formation of smaller lignin fragments (Fig. 1, route b).^9^ However, the formed carbocationic intermediates are highly reactive and can undergo nucleophilic attack by the aromatic structures of other lignin fragments. This interaction leads to repolymerization, forming high-molecular weight lignin structures and stable C–C bonds (Fig. 1, route c).^9^

Carbocation scavengers can be used for capping the reactive carbocation on the lignin backbone and suppress lignin repolymerization (Fig. 1, route d). Wayman *et al.* were among the first to report that the use of specific aromatic compounds as cation scavengers can effectively inhibit lignin repolymerization, thereby enhancing the yield of lignin that is extractable by organic solvents.^10^ More recently, Pielhop *et al.*^11,12^ disclosed that this approach also enhances the bioconversion of cellulose and the chemical value of the obtained lignin. The less repolymerized (or condensed) lignin enhances cellulose accessibility and reduces unproductive cellulase adsorption in enzymatic saccharification. Simultaneously, the lower degree of condensation increases its potential for use in polymers – including a targeted functionalization by the scavenger – or for depolymerization to aromatic building blocks.

2-Naphthol has emerged as one of the most widely used and effective cation scavengers. Its ability to stabilize reactive carbocations can significantly reduce the molecular weight of the resulting lignin.^12–14^ It was further reported that certain monocyclic aromatic compounds, such as dimethylphloroglucinol – which are highly nucleophilic while offering only a single reactive site – demonstrate equal performance in suppressing lignin repolymerization compared to 2-naphthol.^12^ In further studies with monoaromatic compounds, Zhai *et al.*^15^ have shown that additives such as 4-hydroxybenzoic acid, vanillic acid, and syringic acid also act effectively as cation scavengers, improving the enzymatic cellulose saccharification of lignocellulosic biomass. With regard to the valorisation of the lignin fraction, it has recently been confirmed that the use of phenolic compounds such as syringol and phenol allows obtaining an arylated lignin fraction, which is valuable for polymer applications such as the conversion to bisphenols.^16^ These studies show that the use of suitable carbocation scavengers in the (pre)treatment of lignocellulosic biomass can enable improved and holistic utilization of all biomass components.

Previous research revealed that an effective cation scavenger has to provide a nucleophilic active site to react with lignin carbocations and prevent crosslinking of lignin fragments. 2-Naphthol derivatives have shown significant potential due to the high electron density associated with their two aromatic rings, which enhances their ability to stabilize lignin-derived carbocations.^11,17^ In addition, they preferentially undergo only a single aromatic substitution, which prevents lignin crosslinking.^10,12^ More recently, it has been shown that hydroxynaphthoic acid derivatives of 2-naphthol also show excellent performance in repolymerization suppression. Sheng *et al.* reported that 2-hydroxy-1-naphthoic acid, 3-hydroxy-2-naphthoic acid, 6-hydroxy-1-naphthoic acid, and 6-hydroxy-2-naphthoic acid even outperformed 2-naphthol in the dilute acid pretreatment of poplar for enzymatic cellulose saccharification.^18^ Next to that, 3-hydroxy-2-naphthoic acid could also outperform 2-naphthol in the acidified organosolv pretreatment of pine wood for this purpose.^18^ Hydroxynaphthoic acids have generally proven effective for enhancing dilute acid^19–22^ and organosolv^18,20^ pretreatments for the enzymatic saccharification of hardwood,^21,22^ softwood^18,20^ and agricultural residues (wheat straw, corn stover and corn cob).^19^ In those studies, cellulose saccharification yields could be enhanced between 39–72%, compared to a pretreatment without additive.^18,23^ Next to suppressing lignin repolymerization, hydroxynaphthoic acids can also introduce new carboxylic acid groups into the lignin backbone, which decreases lignin hydrophobicity and reduces the non-productive adsorption of cellulase enzymes.^20–23^ It was also found that the addition of hydroxynaphthoic acid increases lignin removal and cellulose accessibility in both dilute acid^20–22^ and in organosolv^20^ pretreatment. Further, pretreatment with hydroxynaphthoic acid also enhanced enzymatic saccharification at high solid loading (10% w/w cellulose), and enabled more than double the ethanol yield in the subsequent fermentation,^20^ demonstrating the high application potential of this approach. However, despite an increasing number of experimental studies on the use of hydroxynaphthoic acids as carbocation scavengers for a range of different biomasses, their different behaviours and activities for preventing lignin repolymerization have not yet been systematically studied and fully understood.

In this work, we propose a synergistic computational and experimental approach to assess the effectiveness of three hydroxynaphthoic acid isomers, namely 3-hydroxy-2-naphthoic acid, 6-hydroxy-1-naphthoic acid, and 6-hydroxy-2-naphthoic acid, as cation scavengers for suppressing lignin repolymerization. Hydroxynaphthoic acids are particularly well suited for this study, since they have already proven as effective scavengers in lignocellulose pretreatment, and since their different isomers allow for the investigation of structure-reactivity relationships in modelling. The three isomers were chosen based on commercial availability and chemical stability in pretreatment. Our findings identify key molecular properties that govern the effectiveness of these scavengers, offering valuable insights for the rational design of novel and even more efficient lignin repolymerization inhibitors.

## 2 Materials and methods

### 2.1 Computational methods

All density functional theory (DFT) calculations presented in this work were performed using the Gaussian 16 software package.^25^ A cluster model of veratrylglycerol-β-guaiacyl ether, (C_18_H_22_O_6_, VG) was employed to represent lignin. The model was constructed by substituting the adjacent lignin structures with a H atom and a methyl capping group, following the approach reported by Mu *et al.*^26^ Geometry optimizations were conducted using the M06-2X level of theory with the 6-311 + G (d,p) basis set.^27^ M06-2X is a meta-hybrid functional with a high percentage of Hartree–Fock exchange, offering improved performance for thermochemistry, noncovalent interactions and barrier heights.^27^ 6-311 + G (d,p) provides a triple-ξ valence description with both polarization and diffuse functions, making it suitable for reaction energetics, noncovalent interactions, and charged intermediates. This combination of functional and basis set has been proved to be accurate enough and suitable for describing the π–π stacking and hydrogen bond interactions involved in lignin molecules.^26^

All atoms were allowed to relax during the optimization to identify the configurations with minimum energy. The binding energy (BE) of scavengers or water with lignin^+^ for all possible configurations was calculated using eqn (1), where *E* indicates the electronic energy:

1 BE = *E*_lignin^+^+molecule_ − *E*_lignin^+^_ − *E*_molecule_ + *E*_BSSE_

Specifically, *E*_lignin^+^+molecule_ is the total energy of lignin^+^ interacting with a molecule (scavenger or water), *E*_lignin^+^_ is the energy of the carbocation intermediate, *E*_molecule_ is the energy of the scavenger or water, and *E*_BSSE_ is the basis set superposition error.

Frequency calculations were conducted at the same level of theory to determine the thermal corrections and identify the transition states.

The binding enthalpy (Δ*H*) was calculated by adding the zero-point (Δ*E*_ZPE_), rotational (Δ*E*_rot_), translational (Δ*E*_trans_), vibrational (Δ*E*_vib_) contributions, and the pressure volume work Δ(PV) to the previously calculated electronic BE, as shown in eqn (2):

2 Δ*H* = BE + Δ*E*_ZPE_ + Δ*E*_rot_ + Δ*E*_trans_ + Δ*E*_vib_ +Δ(PV)

The Gibbs free energy (Δ*G*) was calculated following eqn (3):

3 Δ*G* = Δ*H* − *T*Δ*S*

Specifically, *T* is the temperature (298 K) and Δ*S* is the entropy change associated with the binding step.

Intrinsic reaction coordinate (IRC) calculations were conducted to confirm that the identified transition states smoothly connect to the corresponding reactants and products. In order to investigate the solvent effects, the solvation model based on density (SMD) method was applied for all mentioned calculations by employing water as an implicit solvent, representing the environment of an autohydrolysis or acid pretreatment. Interaction region indicator (IRI) calculations and orbital weighted (OW) Fukui function calculations were conducted at the B3LYP functional level^28^ with the 6-311 + G (d,p) basis set, using Multiwfn software and visualized with VMD packages.^29,30^

### 2.2 Experimental methods

In order to verify the computational predictions of the scavengers' nucleophilicities and reactivities, their bromination rates were compared. Therefore, the protocol for the bromination of aromatic compounds by Carreño *et al.*^31^ was adapted (lower reaction temperature, more diluted solutions, equimolar concentrations of reactants, different solvent, quenching, quantification of unreacted scavenger), in order to enable a kinetic benchmarking assay. 1.0 mM solutions of the hydroxynaphthoic acid isomers and of *N*-bromosuccinimide (NBS) were prepared in acetonitrile (>99.9%), which is a favorable solvent for electrophilic aromatic substitutions. Both stock solutions were pre-cooled for 1 h in the dark in crushed ice prior to mixing. The reaction was initiated by mixing equal volumes (5 mL) of the scavenger solution and the NBS solution in a light-protected and magnetically stirred 10 mL scintillation vial. To enable timely sampling of the highly reactive reactants, the reaction was carried out at 0 °C in an ice bath. 0.5 mL aliquots were taken at different time intervals (15, 30, 60, 120, 300, and 600 s) and immediately quenched in 1.5 mL vials containing an excess of sodium thiosulfate (50 µL of 1% w/v sodium thiosulfate in water mixed with 450 µL acetonitrile). The *t* = 0 s sample was obtained by dispensing 0.25 mL of the NBS solution and 0.25 mL of the scavenger solution directly into the quenching vial. The residual scavenger concentration was quantified by high-performance liquid chromatography (HPLC) as indicated in Table S3. All kinetic runs were performed in duplicate, and single standard deviations are reported with the mean.

In order to study the reactivity of the hydroxynaphthoic acids with real biomass, Norway spruce (*Picea abies*, debarked and milled <1 mm, dry matter content: 92.2%) was pretreated in the presence of either 3-hydroxy-2-naphthoic acid, 6-hydroxy-1-naphthoic acid, or 6-hydroxy-2-naphthoic acid. Pretreatment experiments were carried out in stirred batch reactor vessels (Parr Series 5000 Multiple Reactor System). 2.5 g biomass were mixed with 0.153 g of scavenger (0.205 mol mol^−1^ C_9_ lignin unit) in 40 mL ultrapure water. A control experiment without scavenger was conducted as reference. Reactors were stirred at 400 rpm and heated for 1 h under autogenous pressure at 130, 150, 190 or 210 °C. The 1 h pretreatment time was recorded from the moment the internal reactor temperature reached the target temperature minus 5 °C. At the end of the pretreatment, the reactor vessels were quenched below 50 °C (internal temperature) within 1 min using flowing tap water. The pH of the pretreatment slurry was measured, and 10 mL of the slurry were transferred to 50 mL centrifuge tubes and diluted 1 : 1 (v/v) with methanol to solubilise both unreacted hydroxynaphthoic acids and 2-naphthol that may have formed through degradation. The mixture was vortexed, and 1 mL was filtered through a 0.45 µm PTFE syringe filter (Whatman) for analysis of the hydroxynaphthoic acid and 2-naphthol concentrations by HPLC. All analyses were performed in duplicate. The detailed chromatographic conditions are provided in the SI (Table S3).

To study the thermal stability of the scavengers, each hydroxynaphthoic acid was also subjected to the pretreatment conditions in the absence of biomass. Therefore, ultrapure water was adjusted to pH 3.5 with 0.1 M glacial acetic acid, matching the pH of the biomass slurry at the end of a pretreatment without additive (control). 40 mL of this solution were combined with 0.153 g scavenger, added to the reactor vessel, stirred at 400 rpm and heated for 1 h at 130, 150, 190 or 210 °C under autogenous pressure as described above. Duplicate runs were performed for each scavenger. Work-up and HPLC analysis were identical as described for the experiments with biomass.

The pretreated biomass underwent enzymatic saccharification, to study the scavenger effect on the digestibility of the cellulose fraction. Therefore, pretreatments were carried out at 210 °C as described above, but with a pretreatment time of 2 h to enhance enzymatic conversion. Enzymatic saccharification of the pretreated biomass was conducted according to the National Renewable Energy Laboratory (NREL) standard procedure, preparing 10 g of slurry with a cellulose concentration of 1% w/w in 20 mL scintillation vials.^32^ The following adaptions were made: sodium azide with a final concentration of 0.2 g L^−1^ was used instead of antibiotics, and the pH of the 0.05 M sodium citrate buffer was adjusted to 5.0. Accellerase 1500 (Genencor) with an activity of 26 FPU (filter paper units) mL^−1^, determined according to the NREL standard procedure,^33^ was used with a starting concentration of 15 FPU g^−1^ cellulose. The samples were incubated in a shaker (Multitron Standard; Infors HT) with a shaking throw of 25 mm, at 180 rpm and 50 °C. After 72 h, a second dose of 15 FPU g^−1^ cellulose was added, resulting in a total of 30 FPU g^−1^ cellulose, and the enzymatic saccharification was allowed to proceed further 72 h (in total 144 h). 0.3 mL samples of the biomass slurry were taken after 72 h and 144 h and analysed for sugars in the supernatant by HPLC. All saccharification experiments were carried out in triplicate, and single standard deviations are reported with the mean.

The lignin of the pretreated biomass was also isolated and analysed. Therefore, pretreatments were carried out as described for the enzymatic saccharification experiments. The pretreated biomass was analysed for its cellulose, hemicellulose, acid-insoluble lignin (AIL) and acid-soluble lignin (ASL) contents.^34,35^ The lignin of the biomass was then isolated by enzymatic cellulose saccharification with an excess of cellulase enzymes as described elsewhere,^11^ to remove cellulose as much as possible. Therefore, the pretreated biomass underwent enzymatic saccharification as described above, however with a higher enzyme concentration of 180 FPU g^−1^ cellulose and in 250 mL flasks^32^ for 120 h. The solids from enzymatic hydrolysis were recovered by filtration and centrifugation-washed two times with 15 mL pH 2.5 HCl solution and once with 25 mL pH 9.6 carbonate buffer, using a 50 mL centrifuge tube. Afterwards, the lignin residues were treated with bacterial protease (Type XXIV), in order to remove adsorbed cellulase enzymes.^36,37^ Therefore, the lignin residues were dispensed in carbonate buffer (pH 9.6) at a concentration of 50 mg solids mL^−1^ and a protease concentration of 0.02 mg mg^−1^ lignin. The protease treatment was carried out for 60 h at 37 °C and 200 rpm in a shaker (Multitron Standard; Infors HT). The recovered biomass was centrifuge washed three times with 15 mL pH 2.5 HCl and then freeze dried for 48 h. To enhance lignin extractability, the residues were ball milled (Retsch Planetary Ball Mill PM 400, eight Ø10 mm zirconium oxide balls). Milling was carried out with a low rotation speed of 250 min^−1^ for 20 h (pausing every 5 h for 15 min and changing the rotation direction) to limit heating of the biomass. After milling, the composition of the samples (cellulose hemicellulose, AIL, ASL) was determined.

For nuclear magnetic resonance (NMR) analysis, samples were prepared by extracting 28 mg of the ball-milled residues in 0.75 mL of dimethyl sulfoxide (DMSO)-d6 (Deutero, 99.8% atom% D). Extraction was carried out in 1.5 mL reaction tubes in a shaker (Multitron Standard; Infors HT) at 50 °C for 24 h and 210 rpm. After extraction, the samples were centrifuged and the supernatant used for NMR analysis. The extracted residues were dried overnight at 60 °C, and the extraction yield was determined gravimetrically. ^13^C–^1^H heteronuclear single quantum coherence (HSQC) spectra were recorded at ambient temperature on a Bruker Avance III HD 500 MHz spectrometer using a standard Bruker pulse program.

For size-exclusion chromatography (SEC) analysis, 2 mg of the ball milled residues were extracted with 1 mL of NaOH_aq_ at pH 12 in 1.5 mL reaction tubes using an ultrasonic bath (Elmasonic Easy 100H) for 5 min. The extraction yield of the biomass samples was determined gravimetrically, and lignin concentrations of 0.5–1.2 mg mL^−1^ were obtained. SEC analysis was performed on a Thermo Fisher Vanquish Flex UHPLC System equipped with a cascade of SEC columns from Polymer Standards Service GmbH (MCX combination medium HR, 100–1000000 Da, 2 columns 8 × 300 mm + precolumn) and UV detector (254 nm). The eluent (0.1 mol^−1^ NaOH_aq_.) was freshly prepared, and the columns were operated at 40 °C at a constant flow rate of 1 mL min^−1^. The calibration was performed with 10 poly-(styrenesulfonate) sodium salt standards with low dispersity in the range of 976000–891 g mol^−1^. The retention time of monomeric species in the chromatogram was determined with vanillin.^38^

## 3 Results and discussion

### 3.1 Model construction of VG and cation scavengers

In order to identify the most stable configuration of VG, we optimized four different possible geometries based on the possible conformations permitted by rotation around the C_α_–C_β_ bond and the orientation of hydroxyl group. Fig. S1 shows the optimized molecular structures, along with the corresponding dihedral angle (C–C_α_–C_β_–O) and the relative electronic energy (*E*_relative_). Among the four configurations, VG (a) exhibits the lowest electronic energy, making it the most energetically favourable structure by a margin of 5.9 to 37.3 kJ mol^−1^. As shown in Fig. S1, strong intramolecular H-bonds are present in all optimized structures, occurring either between two hydroxyl groups or between a hydroxyl group and an oxygen atom. Both types of H-bonds can be observed in VG (a) and VG (b), contributing to their enhanced stability compared to the other two structures. VG (a), with the shortest hydrogen bond distances (1.93 Å and 1.99 Å), is the most stable configuration and was therefore selected for subsequent studies.

The representative cation scavengers investigated in the present study include 3-hydroxy-2-naphthoic acid, 6-hydroxy-1-naphthoic acid, and 6-hydroxy-2-naphthoic acid. Various cluster models were considered for each scavenger, based on the orientation and spatial arrangement of their hydroxyl and carboxyl functional groups. Fig. S2 exemplarily shows three optimized structures of 3-hydroxy-2-naphthoic acid, with the corresponding calculated *E*_relative_. The partial positive charge on H(OH) and the localization of negative charge on the double-bonded oxygen of the carboxylic group, facilitates the formation of a hydrogen bond between these two atoms, resulting in a more stable, minimum energy configuration. Fig. S3 shows the selected cluster models constructed for the three cation scavengers investigated in the present study.

**Fig. 2:**
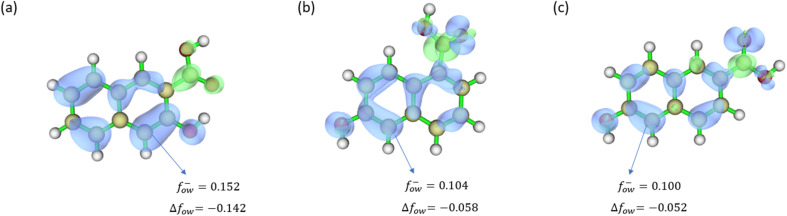
Calculated condensed orbital weighted Fukui function (*f*_ow_^−^) and dual descriptor (Δ*f*_ow_) for the cation scavengers (a) 3-hydroxy-2-naphthoic acid (b) 6-hydroxy-1-naphthoic acid (c) 6-hydroxy-2-naphthoic acid. Light yellow, white and red atoms correspond to C, H and O, respectively. The blue isosurfaces represent the positive Δ*f*_ow_, the green isosurfaces represent the negative Δ*f*_ow_. The blue arrows indicate the most nucleophilic C atoms.

In order to identify the binding site of each scavenger with the lignin framework, we employed the orbital-weighted (OW) Fukui functions and performed dual descriptor calculations for all candidate molecules. These approaches, established within the framework of DFT, are widely used for identifying reactive sites and assessing local reactivity in molecular systems.^39^ The orbital-weighted Fukui functions *f*_ow_^+^ and *f*_ow_^−^ represent the electron density contributions from the LUMO and HOMO, respectively. The dual descriptor (Δ*f*_ow_), defined as the difference between *f*_ow_^+^ and *f*_ow_^−^ (*i.e.*, Δ*f*_ow_ = *f*_ow_^+^ − *f*_ow_^−^), provides insights into local reactivity. In particular, a site with a more positive *f*_ow_^−^ and more negative Δ*f*_ow_ is more susceptible to electrophilic attack. Fig. 2 shows the most positive calculated Fukui function *f*_ow_^−^ and the corresponding most negative dual descriptors. The numerical labels correspond to the highest integrated values of the respective orbital-weighted functions within the Hirshfeld space of each specific atom.

It is worth noting that the identified nucleophilic carbon sites exhibit Δ*f*_ow_ values significantly more negative (35% to 40%) compared to the second most nucleophilic carbon for all selected scavengers. Blue isosurfaces represent regions with negative values of Δ*f*_ow_, while green isosurfaces indicate positive values. Among all atoms in the three analysed scavenger molecules, the C4 nucleophilic carbon site in 3-hydroxy-2-naphthoic acid exhibits a Δ*f*_ow_, of −0.142, as shown in Fig. 2, which is significantly more negative compared to the corresponding values of 6-hydroxy-1-naphthoic acid (−0.058) and 6-hydroxy-2-naphthoic acid (−0.052). This indicates a higher nucleophilicity, suggesting that this carbon site in 3-hydroxy-2-naphthoic acid is more reactive toward an electrophilic species such as lignin^+^, thereby facilitating the formation of the C–C bond between lignin and scavenger. The results of the Fukui function calculations are in excellent agreement with the expected electronic effects of the hydroxyl and carboxylic acid substituents on the naphthalene ring. The hydroxyl group acts as a strong electron-donating substituent through resonance interaction between the oxygen lone pair and the aromatic π system, increasing electron density at the ortho and para positions and therefore enhancing susceptibility to electrophilic attack. In contrast, the carboxylic acid substituent exerts an electron-withdrawing effect, leading to deactivation of the aromatic ring, reduced electron density at the ortho and para positions, and a preference for electrophilic meta-substitution.

In 3-hydroxy-2-naphthoic acid, the synergistic influence of the hydroxyl and carboxylic acid groups leads to an accumulation of electron density at the C4 position, resulting in the highest electron density at this carbon site. By contrast, in both 6-hydroxy-1-naphthoic acid and 6-hydroxy-2-naphthoic acid, the increase in electron density at the C5 position induced by the hydroxyl group is partially counterbalanced by the electron-withdrawing effect of the carboxylic acid group.

### 3.2 Mechanism of VG dehydration

To compare the effectiveness of the different scavengers in suppressing lignin repolymerization, this and the subsequent sections present simulations of the acid-catalyzed lignin dehydration and the electrophilic aromatic substitution for the selected hydroxynaphthoic acids.

**Fig. 3:**
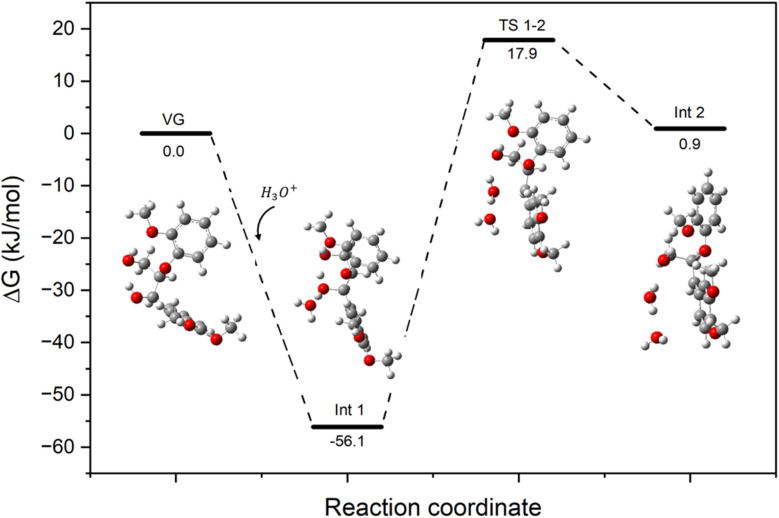
Reaction coordinate diagram showing the Gibbs free energy profile for the acid-catalysed dehydration of VG with schematic representation of key molecular structures. Grey, white and red atoms correspond to C, H, and O, respectively.

Fig. 3 shows the calculated Gibbs free energy profile for the acid-catalysed dehydration of VG. The first elementary step of the reaction pathway is the coordination of a hydronium ion (H_3_O^+^) to the VG molecule *via* formation of a strong H-bond (1.05 Å) between H(H_3_O^+^) and O(VG), which results in the formation of the reaction intermediate Int1. The following dehydration step leads to the formation of a benzylic carbocation intermediate (Int2) *via* a transition state (TS1-2) with an energy barrier of 74.0 kJ mol^−1^. The corresponding enthalpy profile of the VG dehydration pathway is reported in the SI in Fig. S4.

### 3.3. Mechanism of electrophilic aromatic substitution

**Fig. 4:**
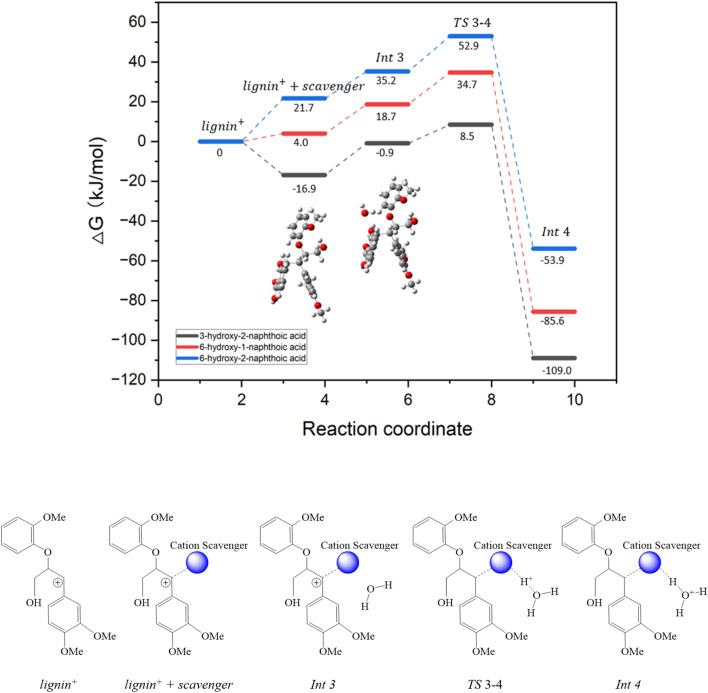
Reaction coordinate diagram showing the Gibbs free energy profile for 3-hydroxy-2-naphthoic acid (black), 6-hydroxy-1-naphthoic acid (red), 6-hydroxy-2-naphthoic acid (blue) with schematic structures for the cation scavenging and corresponding deprotonation of the lignin-scavenger complex. The inset structures show the optimized geometries of lignin^+^ with 3-hydroxy-2-naphthoic acid (left) and Int3 for 3-hydroxy-2-naphthoic acid (right). Grey, white and red atoms correspond to C, H, and O, respectively.

Following its formation, the carbocation intermediate (lignin^+^) interacts with the scavenger through the primary nucleophilic carbon atom identified in the previous section, as depicted in the reaction coordinate diagram in Fig. 4. The following coordination of this complex with a water molecule leads to the formation of intermediate Int3. This species undergoes deprotonation facilitated by the coordinated water molecule *via* the transition state TS3-4, with an associated energy barrier ranging from 9.4 to 17.7 kJ mol^−1^, depending on the hydroxynaphthoic acid isomer. This elementary step results in the formation of Int4. The interaction site between lignin and water molecules is shown in the inset of Fig. 4 corresponding to intermediate Int3. During geometry optimization, the water molecule is stabilized by two hydrogen bonds: one formed between H(H_2_O) and O(lignin^+^), and a second between O(H_2_O) and H(scavenger). Table S1 shows the calculated Gibbs free energy (Δ*G*) for the binding interaction step, ranging between −16.8 to 21.7 kJ mol^−1^ for the three scavengers. By contrast, the Δ*G* between lignin^+^ and the lignin fragment generated during the cleavage of the β-arylether linkages of VG (Fig. 1, route b) was calculated to be 33.3 kJ mol^−1^. The results indicate that all examined scavengers exhibit a stronger binding affinity toward lignin^+^ than the corresponding lignin fragment, demonstrating their potential in preventing lignin repolymerization.

Table S2 shows the calculated enthalpy changes (Δ*H*) associated with the binding interaction of lignin^+^ with the three scavengers and with water. It is worth noting that the binding of lignin^+^ with both the scavengers and water is enthalpically favourable. However, these binding steps are dominated by an entropic penalty which results in a positive Δ*G* for 6-hydroxy-1-naphthoic acid and 6-hydroxy-2-naphthoic acid, as depicted in Fig. 4 (lignin^+^ + scavenger). A comparison of the cluster models for Int3, TS3-4, and Int4 reveals a substantial change in the dihedral angle between the aromatic rings of the scavengers and the lignin^+^ complex, shifting from 28° to 90° as the deprotonation reaction progresses, consistent with trends reported in the literature for similar electrophilic aromatic substitutions.^26^ Despite this pronounced structural variation, the Gibbs free energy barriers associated with the deprotonation step remain modest. It is worth noting that 3-hydroxy-2-naphthoic acid exhibits the lowest deprotonation barrier of (9.4 kJ mol^−1^) in comparison to 6-hydroxy-1-naphthoic acid (16.0 kJ mol^−1^), and 6-hydroxy-2-naphthoic acid (17.7 kJ mol^−1^), as depicted in Fig. 4 (Int3 to TS3-4). The low activation barrier, combined with the favourable binding affinity of 3-hydroxy-2-naphthoic acid toward the lignin^+^ intermediate, identifies this isomer as the most effective scavenger for suppressing lignin repolymerization.

All simulations in this study were performed using an implicit solvation model to simulate the presence of water. To rule out potential artefacts from the solvation approach when analysing scavenger-water binding, binding energy calculations for all selected scavengers were additionally carried out in the gas phase. The results indicate stronger binding between the scavengers and lignin^+^ for 3-hydroxy-2-naphthoic acid (−41.8 kJ mol^−1^), 6-hydroxy-1-naphthoic acid (−25.0 kJ mol^−1^) and 6-hydroxy-2-naphthoic acid (−16.3 kJ mol^−1^). However, the overall binding trend remains consistent.

### 3.4 Lignin-scavenger coordination

To elucidate the lignin-scavenger binding interaction, interaction region indicator (IRI) calculations were applied to the optimized configurations (Fig. S5) following an approach previously reported in the literature.^40^ The isosurfaces reveal the repulsive or attractive forces, including van der Waals (vdW) interactions and hydrogen bonds that exist both between different molecules and among functional groups within the same molecule. The vdW interactions including π–π stacking between aromatic rings, weak hydrogen bonding between H(scavenger) and O(scavenger), and weak hydrogen bonding between H(scavenger) and O(lignin^+^) are indicated by green isosurfaces. The repulsion interactions, such as those occurring in the centre of aromatic rings are represented by red isosurfaces. In addition, relatively strong interactions, primarily hydrogen bonds, are depicted as blue isosurfaces in the IRI map. The scattered map depicted in Fig. S6 quantifies the intensity of the isosurfaces. The points at the base of each peak in Fig. S6 correspond to IRI minima, representing the interaction regions between atoms, as illustrated in Fig. S5. In the IRI analysis, *ρ* indicates the electron density, sign(*λ*_2_) denotes the sign of the second largest eigenvalue of Hessian of *ρ*. Peaks appearing on the negative *x*-axis, where sign(*λ*_2_)*ρ* < 0, indicate strong attractive interactions, which correspond to blue isosurfaces in the IRI analysis. In contrast, peaks on the positive *x*-axis (sign(*λ*_2_)*ρ* > 0) indicate atomic repulsion and are visualized as red isosurfaces. As demonstrated in both Fig. S5 and S6, in addition to the weak interaction between lignin^+^ and the scavengers – which are the primary focus of this study – strong bonds and weak intramolecular interaction within the individual molecules are also observed.

**Fig. 5:**
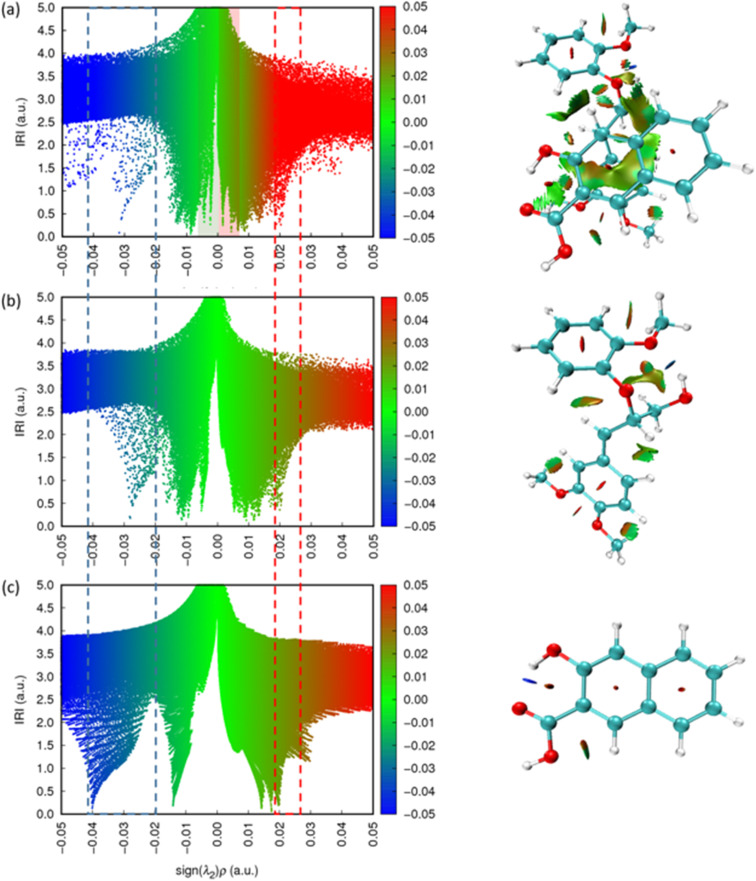
Scatter map of IRI *vs.* sign(*λ*_2_)*ρ* (−0.05 to 0.05) of (a) lignin^+^ and 3-hydroxy-2-naphthoic acid, (b) lignin^+^, (c) 3-hydroxy-2-naphthoic acid (left), and corresponding IRI isosurfaces (right). The blue dash box indicates peaks corresponding to hydrogen bonding and the red dash box indicates peaks corresponding to the repulsion between atoms. The coloured backgrounds in (a) highlight the attractive (green) and repulsive (red) interactions between the scavenger and the lignin carbocation.

Fig. 5 shows the IRI analysis with the corresponding scatter maps (sign(*λ*_2_)*ρ* ranging from −0.05 to 0.05) for the lignin^+^ and 3-hydroxy-2-naphthoic acid complex (a), lignin^+^ (b), and 3-hydroxy-2-naphthoic acid (c), respectively. The peaks with most negative value of sign(*λ*_2_)*ρ*, highlighted in the blue dashed box, correspond to the strongest interactions in the system, specifically, the hydrogen bond between H(lignin^+^) and O(lignin^+^) within the lignin^+^ cation and the hydrogen bond between H(scavenger) and O(scavenger) within the scavengers. Conversely, the peak with the most positive value of sign(*λ*_2_)*ρ*, indicated in the red dashed box represents the repulsive steric interaction in the aromatic rings – an effect consistently observed across all designed systems. In Fig. 5, many overlapping peaks with identical values can be observed in both the individual components (lignin^+^ or scavengers) and the complex. These peaks correspond primarily to vdW interactions and atomic repulsions. By isolating these overlapping regions, we determined that the weak interactions describing the binding between lignin^+^ and the scavengers are predominantly distributed within the range of −0.02 to 0.02. The peaks in the scatter map describing the attractive and repulsive interactions between the scavenger and the lignin carbocation are highlighted with a green and red background, respectively.

**Fig. 6:**
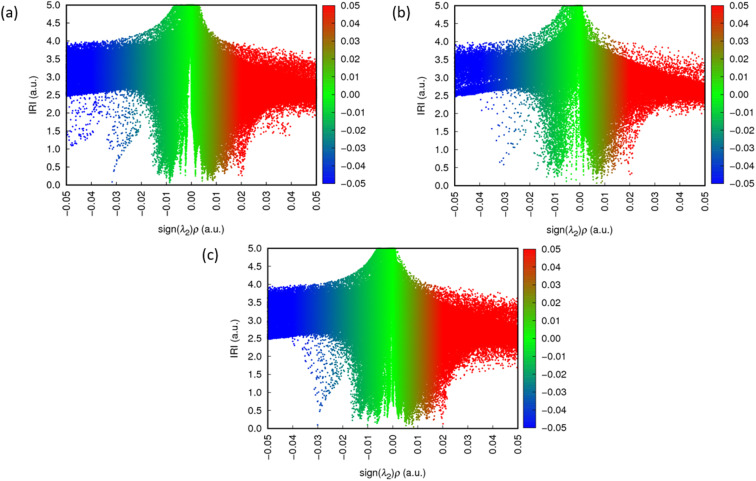
Scatter map of IRI *vs.* sign(*λ*_2_)*ρ* ranging from −0.05 to 0.05 for lignin^+^ coordination with (a) 3-hydroxy-2-naphthoic acid, (b) 6-hydroxy-1-naphthoic acid and (c) 6-hydroxy-2-naphthoic acid.

Fig. 6 presents the scatter maps describing the interaction of the lignin carbocation with the different scavengers. The binding interactions between 3-hydroxy-2-naphthoic acid and lignin^+^ are described by a highly intense peak concentrated in a restricted interval around sign(*λ*_2_)*ρ* = −0.01. On the other hand, the attractive interactions between lignin, 6-hydroxy-1-naphthoic acid and 6-hydroxy-2-naphthoic acid are described by multiple and relatively dispersed peaks. This analysis provides a rationale for the stronger binding between 3-hydroxy-2-naphthoic acid and lignin^+^ compared to the other investigated isomers, presented in Fig. 4.

### 3.5 Experimental validation

**Fig. 7:**
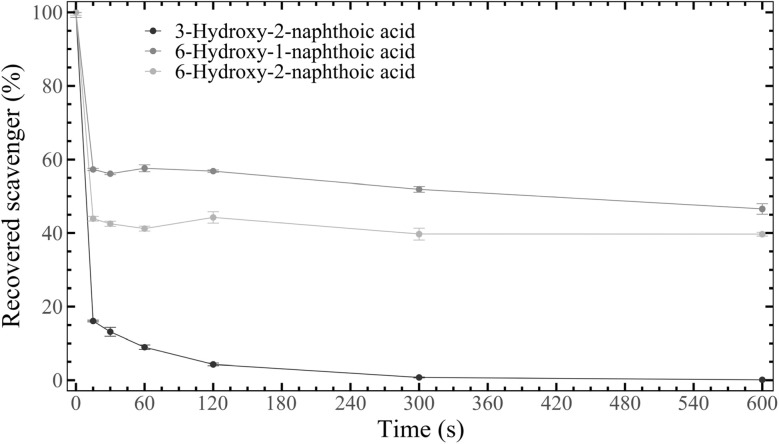
Kinetics of the bromination of the hydroxynaphthoic acids with NBS, showing the residual concentrations of 3-hydroxy-2-naphthoic acid, 6-hydroxy-1-naphthoic acid and 6-hydroxy-2-naphthoic acid. Experimental conditions: 0.5 mM hydroxynaphthoic acid, 0.5 mM NBS, temperature 0 °C.

To provide experimental evidence for the reactivity trends predicted by the Fukui dual descriptors (Fig. 3), the bromination rates of the hydroxynaphthoic acids were compared. NBS was used as brominating agent, serving as an electrophilic bromine source, which enables benchmarking the hydroxynaphthoic acids' propensity for electrophilic substitution. Bromination was carried out under equimolar conditions with very diluted solutions and at reduced temperature, in order to enable a kinetic benchmarking assay. Nevertheless, most of the reaction occurred within the first 15 s, with the hydroxynaphthoic acids exhibiting different reactivities (Fig. 7). 3-Hydroxy-2-naphthoic acid was the most reactive compound, reaching a conversion of 83.9% after 15 s. In contrast, 6-hydroxy-1-naphthoic acid and 6-hydroxy-2-naphthoic acid reacted substantially more slowly, achieving conversions of only 42.7% and 56.1%, respectively, within 15 s. The observed differences are largely determined by the reaction kinetics in the initial phase, whereas changes in the concentration of the hydroxynaphthoic acids after 15 s were comparatively small. The higher and faster conversion of 3-hydroxy-2-naphthoic acid compared to 6-hydroxy-1-naphthoic acid and 6-hydroxy-2-naphthoic acid is consistent with their predicted nucleophilicities and reactivities (Fig. 3). The near-quantitative depletion of 3-hydroxy-2-naphthoic acid under equimolar conditions with NBS suggests substitution dominated by a highly activated site, whereas the partial conversions observed for the 6-hydroxy isomers reflect slower substitution kinetics and could be influenced by competing multi-bromination pathways. Thus, the results provide empirical support for the scavengers' inherent reactivities and are consistent with the computational predictions.

In order to validate the results from the modelling of the lignin-scavenger interaction, autohydrolysis pretreatment experiments were carried out with biomass in the presence of the scavengers. During the autohydrolysis pretreatment, organic acids such as acetic acid are released from the hemicellulose in the biomass,^41^ leading to acidic conditions, which induce lignin repolymerization. However, the addition of carbocation scavengers can effectively suppress the undesired repolymerization.^10^ To study the effectiveness of the modeled scavengers, spruce wood was pretreated with either 3-hydroxy-2-naphthoic acid, 6-hydroxy-1-naphthoic acid, or 6-hydroxy-2-naphthoic acid. The scavenger concentration of 0.205 mol mol^−1^ C_9_ lignin unit allows observing a pronounced effect of aromatic additives in carbocation scavenging.^11,12^ In addition, softwood such as spruce is particularly prone to lignin repolymerization,^11^ and thus well suited for studying the scavenging effect of the hydroxynaphthoic acids. Pretreatment was performed at four different temperatures (130 °C, 150 °C, 190 °C, 210 °C) to span a broad range from low to high scavenger consumption.

**Fig. 8:**
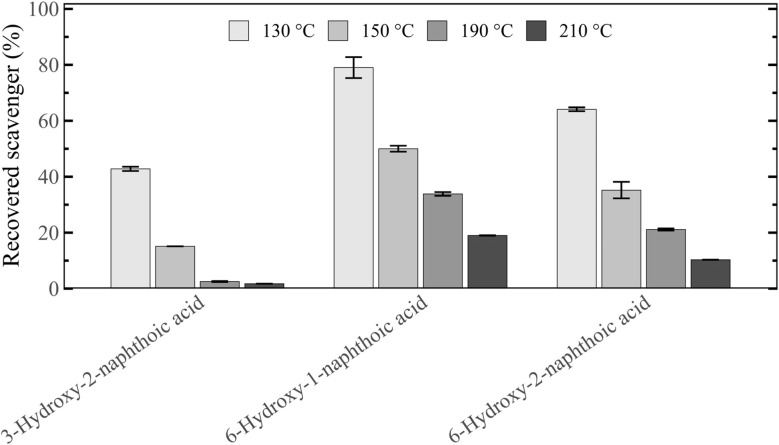
Recovery of the different hydroxynaphthoic-acid scavengers after 1 h autohydrolysis pretreatment of spruce at 130, 150, 190 and 210 °C.

To rank the scavengers' different reactivities with lignin, their residual concentrations after pretreatment in the recovered slurry was analysed (Fig. 8). For all three scavengers, increasing the pretreatment temperature led to a decrease of recovered scavenger, indicating enhanced reaction with lignin and the integration of the scavenger into the solid biomass structure. 3-Hydroxy-2-naphthoic acid was found to be the most reactive compound, followed by 6-hydroxy-2-naphthoic acid and 6-hydroxy-1-naphthoic acid. At 210 °C only 2% of the initially added 3-hydroxy-2-naphthoic acid were recovered after pretreatment, highlighting its high reactivity with lignin. This compares to a much higher recovery of 19% for 6-hydroxy-1-naphthoic acid and a 10% recovery for 6-hydroxy-2-naphthoic acid at the same temperature. The order of reactivity of the three hydroxynaphthoic acids was the same for all four tested temperatures. The observed order is consistent with the Δ*G* trend predicted in Section 3.3 (Fig. 4), indicating that the modelling results are in good agreement with the experimental observations.

To ensure that the observed scavenger recovery after pretreatment was not influenced by a potential degradation of the additives, the hydroxynaphthoic acids were subjected to similar conditions as in pretreatment, but in the absence of biomass. Under these conditions, any decrease in recovery can be attributed solely to degradation processes, such as thermal decarboxylation of hydroxynaphthoic acids to 2-naphthol. To simulate the acidity in the autohydrolysis pretreatment with biomass, the hydroxynaphthoic acids were subjected to a treatment in acidified water adjusted to pH 3.5, matching the pH of the recovered slurry after a pretreatment without additive (control). The treatment time and temperatures were identical to the experiments with biomass (1 h at 130, 150, 190 and 210 °C).

**Fig. 9:**
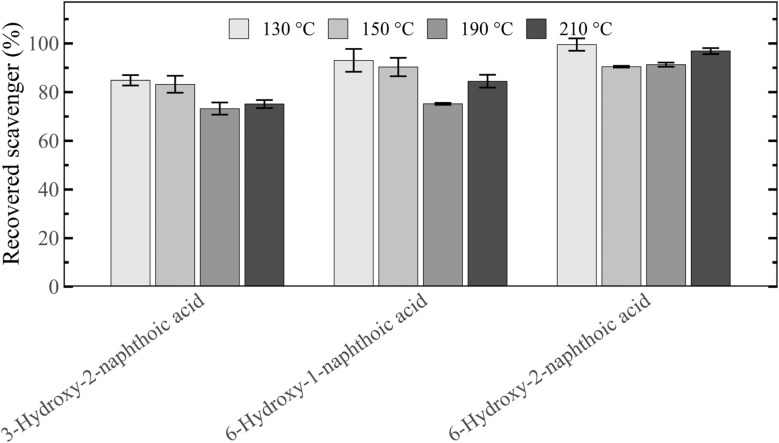
Recovery of the different hydroxynaphthoic-acid scavengers after a 1 h aqueous treatment at 130, 150, 190 and 210 °C at pH 3.5. Experiments were conducted without biomass to analyse potential degradation of the hydroxynaphthoic acids.

In the experiments, between 73–99% of the hydroxynaphthoic acids were recovered (Fig. 9), indicating their relatively high stability under the studied conditions. In addition, only 0–2% of the hydroxynaphthoic acids were recovered as 2-naphthol after the treatment, demonstrating that their thermal decarboxylation to 2-naphthol was minimal. These results confirm that the observed scavenging effect of the hydroxynaphthoic acids cannot be attributed to the *in situ* formation of 2-naphthol. Most importantly, the results confirm that the reduced recovery of the hydroxynaphthoic acids in the experiments with biomass (Fig. 8) is almost exclusively due to the reaction with the biomass and not related to thermal degradation. For example, at 210 °C only 2% of 3-hydroxy-2-naphthoic acid were recovered in the experiment with biomass (Fig. 8), whereas 75% were recovered in the experiment without biomass (Fig. 9). The difference in recovery can be attributed to the reaction with lignin present in the biomass.

It is worth noting that we also conducted experiments with a fourth hydroxynaphthoic acid, namely 2-hydroxy-1-naphthoic acid. However, in the stability trials in acidified water, 2-hydroxy-1-naphthoic acid was practically completely degraded. After treatment at the lowest temperature of 130 °C, only 0.6% of the 2-hydroxy-1-naphthoic acid remained. 95.5% of the compound was recovered as 2-naphthol, showing that 2-hydroxy-1-naphthoic acid is highly susceptible to decarboxylation. Due to its instability, 2-hydroxy-1-naphthoic acid was not included in our studies.

**Fig. 10:**
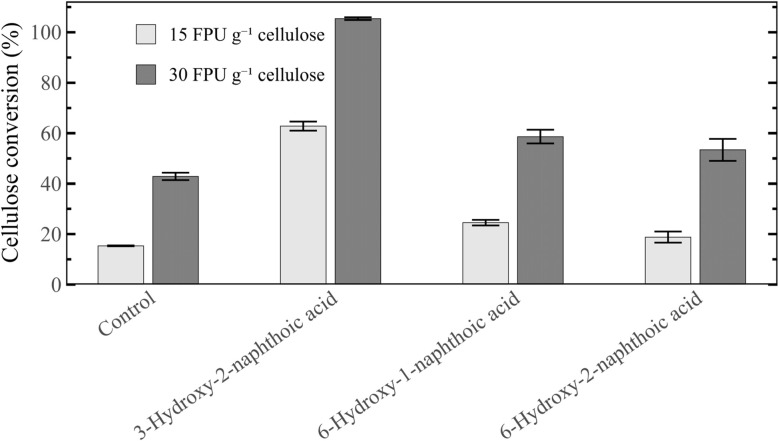
Cellulose conversion (glucose yield) in the enzymatic saccharification of spruce pretreated with 3-hydroxy-2-naphthoic acid, 6-hydroxy-1-naphthoic acid and 6-hydroxy-2-naphthoic acid. An experiment without pretreatment additive (control) is shown for comparison. Saccharification: 1% w/w cellulose, 0–72 h: 15 FPU g^−1^ cellulose, 72–144 h: 30 FPU g^−1^ cellulose. Conversion results are shown for 72 h (15 FPU g^−1^ cellulose) and 144 h (30 FPU g^−1^ cellulose).

The pretreated biomass underwent enzymatic cellulose saccharification, to investigate the scavenger effect on its digestibility. All hydroxynaphthoic acids allowed to increase the glucose yield compared to the control, both at enzyme concentrations of 15 and 30 FPU g^−1^ cellulose (Fig. 10). However, by far the highest glucose yield was obtained with 3-hydroxy-2-naphthoic acid, which enhanced the yield compared to the control by up to 308% (15 FPU g^−1^ cellulose). In particular, it also improved the yield by 156–233% (15 FPU g^−1^ cellulose) compared to the other hydroxynaphthoic acids. This once again confirms its higher reactivity for suppressing lignin repolymerization reactions, since lignin repolymerization is one of the main factors inhibiting enzymatic cellulose conversion.^11^ It is noteworthy that with 30 FPU g^−1^ cellulose, 3-hydroxy-2-naphthoic acid even enabled the complete saccharification of cellulose, which is remarkable for the recalcitrant softwood biomass. To the best of our knowledge, there is only one study in which different hydroxynaphthoic acids were compared in lignocellulose pretreatment. Sheng *et al.* investigated the suppression of lignin repolymerization in the dilute acid pretreatment of beech wood, using 2-hydroxy-1-naphthoic acid, 3-hydroxy-2-naphthoic acid, 6-hydroxy-1-naphthoic acid and 6-hydroxy-2-naphthoic acid as cation scavengers.^23^ Compared to the other hydroxynaphthoic acids, 3-hydroxy-2-naphthoic acid showed a significantly improved performance for enhancing enzymatic cellulose conversion. These experimental results align well with our experimental and simulation findings, providing further validation for our study.

In order to analyse the lignin obtained from biomass pretreatment with the different hydroxynaphthoic acids, additional experiments were conducted. The analysis of the pretreated biomass compositions is shown in Table S4. Hemicellulose was practically completely removed from the biomass due to acidic degradation, and no major differences can be observed among the samples. The lignin content, *i.e.*, the sum of AIL and ASL, increased after treatment with the hydroxynaphthoic acids. Both the increase in AIL and ASL content can be attributed to the integration of carbocation scavengers into the lignin backbone,^11,12^ further confirming that the hydroxynaphthoic acids reacted with the lignin. The biomass treated with 3-hydroxy-2-naphthoic acid exhibited the highest total lignin content (ASL + AIL), highlighting its superior reactivity compared to the other scavengers.

The lignin was further analysed by HSQC NMR spectroscopy. Therefore, the lignin in the pretreated biomass was first isolated by enzymatic cellulose saccharification with an excess of cellulase enzymes, allowing to practically completely remove the cellulose (cellulose content <10%). The biomass residues were additionally ball-milled to achieve higher extraction yields of the different lignins. In the extraction with DMSO, all scavenger additives enhanced the yield of extractable lignin compared to the control (Table S5), confirming their effectiveness in suppressing lignin repolymerization.^10,11^ In addition, the signals observed in the 7–8 ppm (^1^H chemical shift) and 120–130 ppm (^13^C chemical shift) region of the NMR spectra (Fig. S7) provide evidence of naphthalene structure incorporation into the lignin^14^ by the different hydroxynaphthoic acids. Once more, this effect is especially pronounced in the sample pretreated with 3-hydroxy-2-naphthoic acid.

The suppression of lignin repolymerization by the hydroxynaphthoic acids was additionally confirmed through SEC analysis of the isolated lignin residues (Table S6). All hydroxynaphthoic acids resulted in lignins with significantly lower molecular weight and narrower polydispersity index (PDI), which can be attributed to the prevention of lignin condensation (Fig. 1, route b and c). Compared to the control, the weight average molecular weight (Mw) was 6–8 times lower, while the PDI was approximately half as low. Surprisingly, the molecular weight of the 3-hydroxy-2-naphthoic acid sample was slightly increased compared to the 6-hydroxy-1-naphthoic acid and 6-hydroxy-2-naphthoic acid samples. This observation needs further elucidation, but may be related to the fact that effective scavengers can also suppress lignin depolymerization (Fig. 1, route b), next to suppressing repolymerization. Notably, the less condensed lignins with additional aryl functionalisation by the scavenger can have a higher application potential, *e.g.* for the use in polymers or for their depolymerization to aromatic building blocks.^11,12^

## 4 Conclusion

This integrated computational and experimental study provides a comprehensive analysis of three hydroxynaphthoic acid isomers to assess their potential as cation scavengers for the suppression of lignin repolymerization. We characterised and simulated the cation scavenging process with the goal of identifying key molecular descriptors for the rational identification and design of promising scavenger candidates. The analysis of the Fukui functions indicate that all selected isomers possess a predominant nucleophilic site. Among the evaluated scavengers, 3-hydroxy-2-naphthoic acid exhibits the most nucleophilic active site, with a dual descriptor Δ*f*_ow_ = −0.142.

Our results show that the binding energy difference between the scavengers and lignin^+^ is a key descriptor to characterize the performance of the scavenger. As demonstrated by the Fukui function analysis, 3-hydroxy-2-naphthoic acid exhibits the strongest binding energy with lignin^+^ of −16.8 kJ mol^−1^. The calculated binding energy depends on the strength of hydrogen bonding, vdW interactions, and repulsion forces existing between lignin^+^ and the different scavengers. Results from the IRI calculations reveal that the C–C bond formed between the electrophilic carbon of lignin^+^ and the nucleophilic carbon site of the scavenger exhibit comparable strength across all candidates. Consequently, the observed differences in binding energy are primarily driven by the balance between van der Waals interactions and repulsive forces. Among the evaluated compounds, 3-hydroxy-2-naphthoic acid demonstrates the strongest binding affinity with lignin^+^, highlighting its effectiveness as a cation scavenger for the investigated process.

Our experimental results provided further validation for the computational results. Among the evaluated hydroxynaphthoic acids, 3-hydroxy-2-naphthoic acid exhibits the highest reactivities with lignin. Only 2% of 3-hydroxy-2-naphthoic acid were recovered after biomass pretreatment at 210 °C. When investigating the scavenger effect on the digestibility of pretreated lignocellulose, 3-hydroxy-2-naphthoic acid also exhibits the highest glucose yield (up to 308% enhancement compared to the control), which further proves the better performance in preventing lignin repolymerization compared to the other hydroxynaphthoic acids.

To the best of our knowledge, this is the first study to systematically combine the modelling and experimental investigation of carbocation scavengers in lignocellulose pretreatment. The innovation lies not in the examination of hydroxynaphthoic acids as pretreatment additives, but in opening up a completely new route based on computational modelling for studying the effectiveness of carbocation scavengers. Modelling can serve as a powerful starting point for the digital screening of a wide range of compounds, allowing only the most promising candidates to be selected for subsequent laboratory investigation. In this way, new effective compounds can be identified with reduced experimental effort. This represents a conceptual step forward, as it shifts the approach from a largely empirical screening toward a more rational model-guided strategy. In a subsequent stage, modelling can also be used to optimize the general pretreatment conditions. The effects of carbocation scavengers on enhancing lignocellulose pretreatment are case-specific, depending on scavenger, biomass, and pretreatment method.^42^ Consequently, predictive modelling of the corresponding processes can provide insights into reaction pathways and peformance trends.

Future work will focus on extending the approach to a broader range of scavengers and further refining the methodology to enhance predictive power and practical applicability. The further development of this approach may even integrate the use of other digital tools such as AI and data mining, providing foundation for an even more systematic and accelerated progress in this research area. Additionally, considering that hydroxynaphthoic acids are predominantly fossil-derived, these efforts will aid in identifying alternative scavengers that can be sustainably sourced from renewables.

## Conflicts of interest

T. P. is co-inventor of two granted patents on the use of carbocation scavengers in the pretreatment of lignocellulosic biomass to improve the saccharification of cellulose and to produce a lignin fraction for improved utilization in the manufacturing of monomers/polymers (EP11008860.6, US10370501B2).

## Supplementary Material

SU-004-D5SU00841G-s001

## Data Availability

The data supporting this article, including the optimized geometries for the molecular configurations shown in Fig. 2, 4, 5, S1, and S2 are available through the University of Southampton Institutional Repository at https://eprints.soton.ac.uk/503893/. Additional data supporting this article have been included as part of the supplementary information (SI). Supplementary information is available. See DOI: https://doi.org/10.1039/d5su00841g.

## References

[cit1] Wu Y., Ge S., Xia C., Mei C., Kim K.-H., Cai L., Smith L. M., Lee J., Shi S. Q. (2021). Application of intermittent ball milling to enzymatic hydrolysis for efficient conversion of lignocellulosic biomass into glucose. Renew. Sustain. Energy Rev..

[cit2] Chen J., Fan X., Zhang L., Chen X., Sun S., Sun R. C. (2020). Research Progress in Lignin-Based Slow/Controlled Release Fertilizer. ChemSusChem.

[cit3] Li H., Liang Y., Li P., He C. (2020). Conversion of biomass lignin to high-value polyurethane: A review. J. Bioresour. Bioprod..

[cit4] Broda M., Yelle D. J., Serwanska K. (2022). Bioethanol Production from Lignocellulosic Biomass-Challenges and Solutions. Molecules.

[cit5] Brethauer S., Studer M. H. (2015). Biochemical Conversion Processes of Lignocellulosic Biomass to Fuels and Chemicals - A Review. Chimia.

[cit6] Yuan Y., Jiang B., Chen H., Wu W., Wu S., Jin Y., Xiao H. (2021). Recent advances in understanding the effects of lignin structural characteristics on enzymatic hydrolysis. Biotechnol. Biofuels.

[cit7] Solarte-Toro J. C., Romero-García J. M., Martínez-Patiño J. C., Ruiz-Ramos E., Castro-Galiano E., Cardona-Alzate C. A. (2019). Acid pretreatment of lignocellulosic biomass for energy vectors production: A review focused on operational conditions and techno-economic assessment for bioethanol production. Renew. Sustain. Energy Rev..

[cit8] Lundquist K., Lundgren R., Danielsen J., Haaland A., Svensson S. (1972). Acid degradation of lignin. Acta Chem. Scand..

[cit9] Sarkanen K. V. and Ludwig C. H., Lignins: Occurrence, Formation, Structure and Reactions, 1971

[cit10] Wayman M., JH L. (1978). Aspen autohydrolysis: the effects of 2-naphthol and other aromatic compounds. Tappi.

[cit11] Pielhop T., Larrazábal G. O., Studer M. H., Brethauer S., Seidel C.-M., Rudolf von Rohr P. (2015). Lignin repolymerisation in spruce autohydrolysis pretreatment increases cellulase deactivation. Green Chem..

[cit12] Pielhop T., Larrazábal G. O., Rudolf von Rohr P. (2016). Autohydrolysis pretreatment of softwood – enhancement by phenolic additives and the effects of other compounds. Green Chem..

[cit13] Lora J. H., Wayman M. (1980). Simulated autohydrolysis of aspen milled wood lignin in the presence of aromatic additives. Changes in molecular weight distribution. J. Appl. Polym. Sci..

[cit14] Li J., Gellerstedt G. (2008). Improved lignin properties and reactivity by modifications in the autohydrolysis process of aspen wood. Ind. Crops Prod..

[cit15] Zhai R., Hu J., Saddler J. N. (2018). Minimizing cellulase inhibition of whole slurry biomass hydrolysis through the addition of carbocation scavengers during acid-catalyzed pretreatment. Bioresour. Technol..

[cit16] Li N., Yan K., Rukkijakan T., Liang J., Liu Y., Wang Z., Nie H., Muangmeesri S., Castiella-Ona G., Pan X. (2024). Selective lignin arylation for biomass fractionation and benign bisphenols. Nature.

[cit17] Wayman M., Lora J. H. (1980). Simulated autohydrolysis of aspen milled wood lignin in the presence of aromatic additives: Structural
modifications. J. Appl. Polym. Sci..

[cit18] Sheng Y., Liu M., Xia C., Song J., Ge S., Cai L., Lam S. S., Sonne C. (2021). Chem. Eng. J..

[cit19] Liu M., Zuo S., Liang Y., Sheng Y., Ge S., Wu J., Ma H., Sun F., Ahamad T., Van Le Q. (2022). The influence of 3-hydroxy-2-naphthoic acid on agricultural wastes extracted sugar production used as energy sources. Fuel.

[cit20] Xie X., Song K., Wang J., Hu J., Wu S., Chu Q. (2024). Efficient ethanol production from masson pine sawdust by various organosolv pretreatment and modified pre-hydrolysis simultaneous saccharification and fermentation. Renewable energy.

[cit21] Zhang S., Xu X., Li Y., Xie X., Hu J., Wu S., Song K., Chu Q. (2024). Full utilization of poplar sawdust with chemical-mechanical pretreatment for coproduction of xylooligosaccharides, fermentable sugars and porous carbon materials. Ind. Crops Prod..

[cit22] Tan X., Wu X., Wang W., An J., Zhang Q., Tang S., He B., Lai C., Sheng Y. (2025). Effect of nucleophilic additives on phosphoric acid pretreatment of lignocelluloses. Biotechnol. Prog..

[cit23] Sheng Y., Liu M., Xia C., Song J., Ge S., Cai L., Lam S. S., Sonne C. (2021). Using nucleophilic naphthol derivatives to suppress biomass lignin repolymerization in fermentable sugar production. Chem. Eng. J..

[cit24] Pielhop T. (2023). Suppression of Lignin Repolymerisation to Enhance Cellulose Bioconversion and Lignin Valorisation - A Review. Chimia.

[cit25] Gaussian 16 Rev. C.01, Wallingford, CT, 2016

[cit26] Mu X., Han Z., Liu C., Zhang D. (2019). Mechanistic Insights into Formaldehyde-Blocked Lignin Condensation: A DFT Study. J. Phys. Chem. C.

[cit27] Zhao Y., Truhlar D. G. (2007). The M06 suite of density functionals for main group thermochemistry, thermochemical kinetics, noncovalent interactions, excited states, and transition elements: two new functionals and systematic testing of four M06-class functionals and 12 other functionals. Theor. Chem. Acc..

[cit28] Tirado-Rives J., Jorgensen W. L. (2008). Performance of B3LYP density functional methods for a large set of organic molecules. J. Chem. Theor. Comput..

[cit29] Lu T., Chen F. (2012). Multiwfn: A multifunctional wavefunction analyzer. J. Comput. Chem..

[cit30] Lu T. (2024). A comprehensive electron wavefunction analysis toolbox for chemists, Multiwfn. J. Chem. Phys..

[cit31] Carreno M. C., Garcia Ruano J. L., Sanz G., Toledo M. A., Urbano A. N.- (1995). Bromosuccinimide in acetonitrile: A mild and regiospecific nuclear brominating reagent for methoxybenzenes and naphthalenes. J. Org. Chem..

[cit32] Selig M. , Weiss N., Ji Y., Enzymatic Saccharification of Lignocellulosic Biomass Technical Report No; NREL/TP-510-42629, 2008

[cit33] Adney B. and Baker J., Measurement of cellulase activities, NREL/TP-510-42628, National Renewable Energy Laboratory, Golden, CO, 2008

[cit34] Sluiter A. , Hames B., Ruiz R., Scarlata C., Sluiter J., Templeton D. and Crocker D., National Renewable Energy Laboratory, Golden, 2008

[cit35] Hames B. , Ruiz R., Scarlata C., Sluiter A., Sluiter J. and Templeton D., Preparation of Samples for Compositional Analysis, Technical Report NREL/TP-510-42620, National Renewable Energy Laboratory, 2008, 1 9

[cit36] Rahikainen J., Mikander S., Marjamaa K., Tamminen T., Lappas A., Viikari L., Kruus K. (2011). Inhibition of enzymatic hydrolysis by residual lignins from softwood—study of enzyme binding and inactivation on lignin-rich surface. Biotechnol. Bioeng..

[cit37] Argyropoulos D. S. , Advances in Lignocellulosics Characterization, 1999

[cit38] Voitl T., Nagel M. V., Rudolf von Rohr P. (2010). Analysis of products from the oxidation of technical lignins by oxygen and H3PMo12O40 in water and aqueous methanol by size-exclusion chromatography. Holzforschung.

[cit39] Pino-Rios R., Inostroza D., Cardenas-Jiron G., Tiznado W. (2019). Orbital-Weighted Dual Descriptor for the Study of Local Reactivity of Systems with (Quasi-) Degenerate States. J. Phys. Chem. A.

[cit40] Li C., Vernuccio S., Moghadam P. Z. (2025). Computational screening of amino-functionalized molecules for direct air capture of CO2. J. Phys. Chem. A.

[cit41] Mosier N., Wyman C., Dale B., Elander R., Lee Y., Holtzapple M., Ladisch M. (2005). Features of promising technologies for pretreatment of lignocellulosic biomass. Bioresour. Technol..

[cit42] Nikqi B., Salzmann J., Eckl M. A., Di Francesco D., Pielhop T. (2025). Carbocation scavenger enhanced pretreatment of wood for enzymatic saccharification-Experimental study and review. Ind. Crops Prod..

