# Supplementary material for: Mechanistic insights into hydroxynaphthoic acid-based suppression of lignin repolymerization
Source: RSC Sustain. 2026 Apr 13;4(5):2396–409. doi: 10.1039/d5su00841g (PMC13094718; doi:10.1039/d5su00841g)
Supplement: SU-004-D5SU00841G-s001 [file SU-004-D5SU00841G-s001.pdf]

This journal is © The Royal Society of Chemistry 2026

Supporting Information

Mechanistic Insights into Hydroxynaphthoic Acid–Based Suppression of Lignin Repolymerization

Chenhao Li†, Matthias Alexander Ulrich Eckl⁂, Pascal Fitz⁂, Thomas Pielhop⁂, Sergio Vernuccio‡**

† School of Chemical, Materials and Biological Engineering, The University of Sheffield, Sheffield, United Kingdom.

‡ School of Chemistry and Chemical Engineering, University of Southampton, Southampton, United Kingdom.

⁂ Institute of Chemistry and Biotechnology, Zurich University of Applied Sciences, Zurich, Switzerland.

*Corresponding authors: Sergio Vernuccio (s.vernuccio@soton.ac.uk), Thomas Pielhop (thomas.pielhop@zhaw.ch)

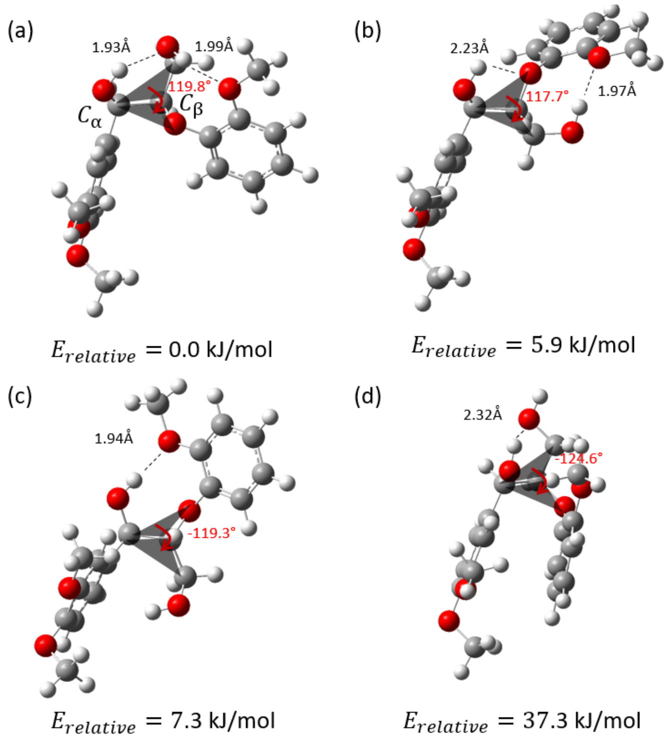

Figure S1. Different optimized configurations of VG. Grey, white and red atoms correspond to C, H, and O, respectively. The red numbers represent the dihedral angles C–C_α–C_β–O in degrees (°). The dashed lines represent the distance between pairs of atoms identified as the primary interaction sites in angstroms (Å). The numbers below each configuration represent the relative electronic energy ($E_{relative}$).

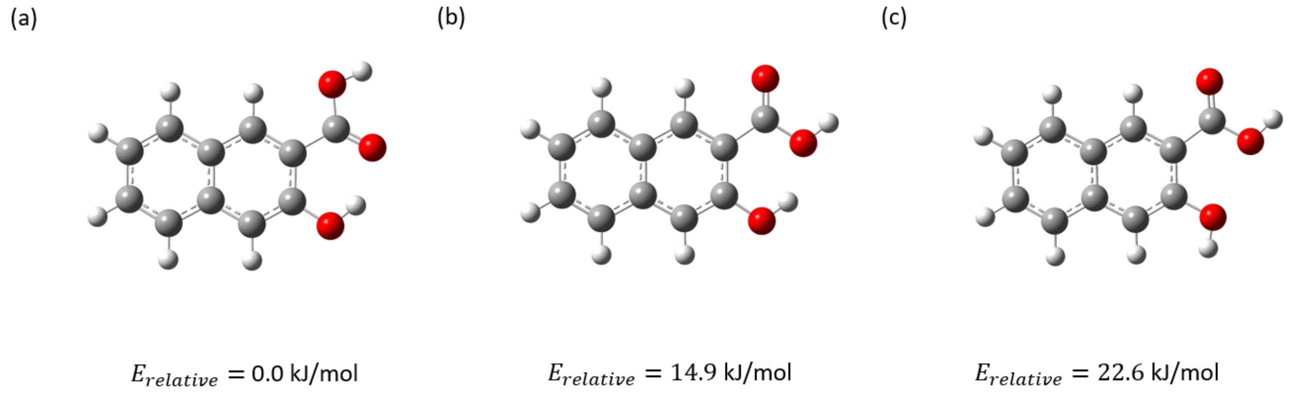

Figure S2. Different optimized configurations of a scavenger, illustrated for 3-hydroxy-2-naphthoic acid as an example. Grey, white and red atoms correspond to C, H and O, respectively. The numbers below each configuration represent the relative electronic energy ($E_{relative}$).

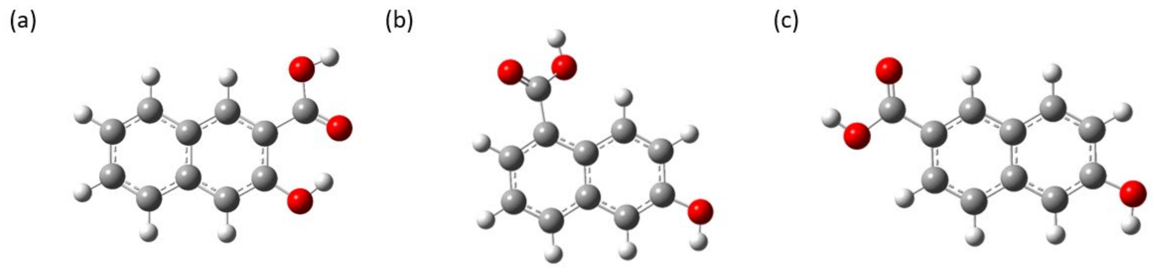

Figure S3. Constructed cluster models of selected cation scavengers (a) 3-hydroxy-2-naphthoic acid, (b) 6-hydroxy-1-naphthoic acid, and (c) 6-hydroxy-2-naphthoic acid. Grey, white and red atoms correspond to C, H and O, respectively.

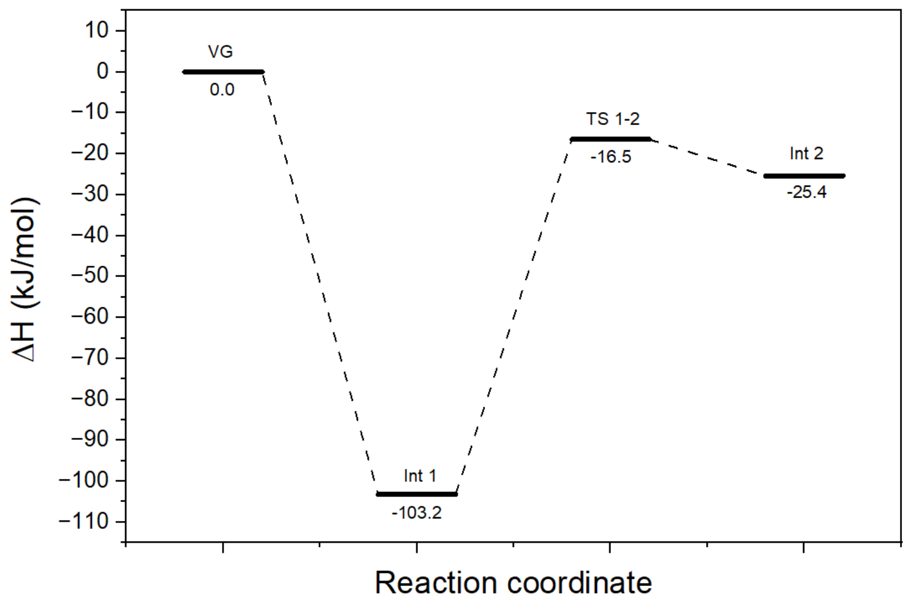

Figure S4. Reaction coordinate diagram showing the thermal enthalpy profiles for the acid-catalysed dehydration of VG.

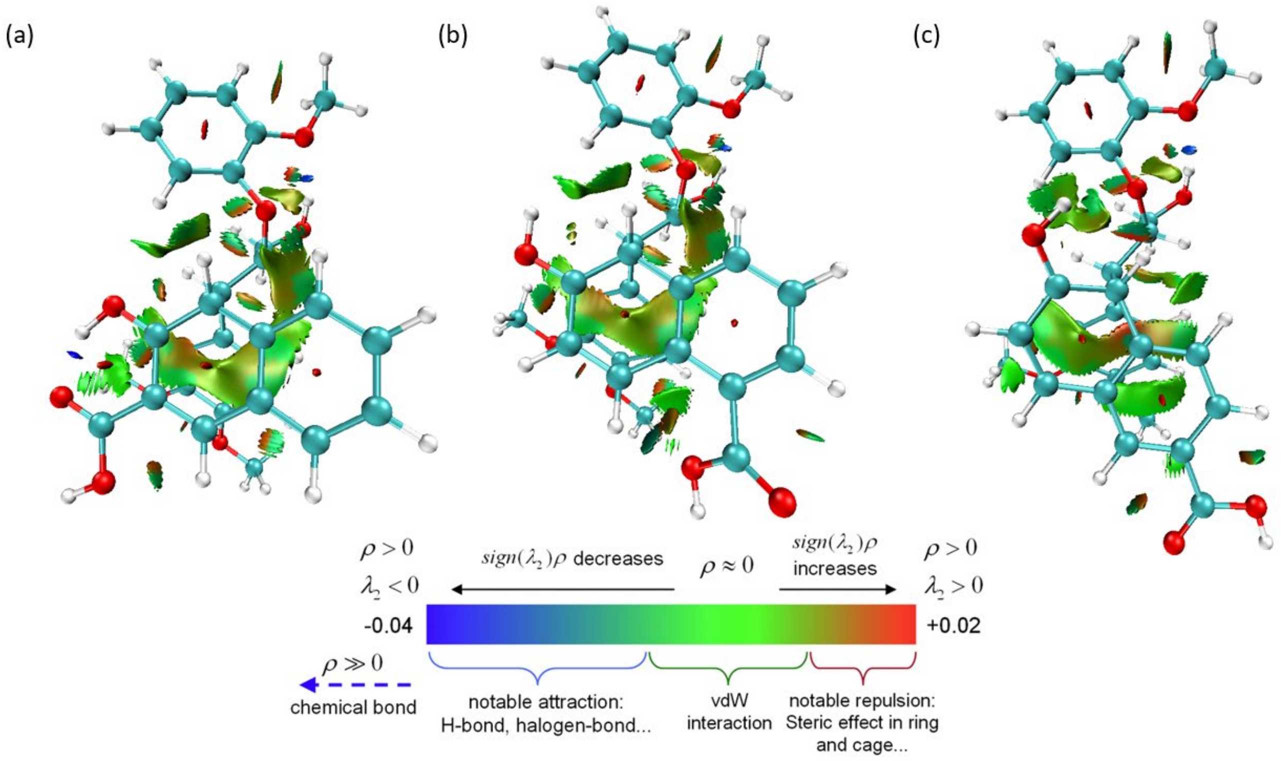

Figure S5. IRI calculations for $lignin^+$ binding with (a) 3-hydroxy-2-naphthoic acid, (b) 6-hydroxy-1-naphthoic acid and (c) 6-hydroxy-2-naphthoic acid at IRI = 1.0. Red, turquoise, white atoms correspond to O, C and H, respectively. ρ indicates the electron density, $sign(\lambda_2)$ denotes the sign of the second largest eigenvalue of Hessian of ρ.

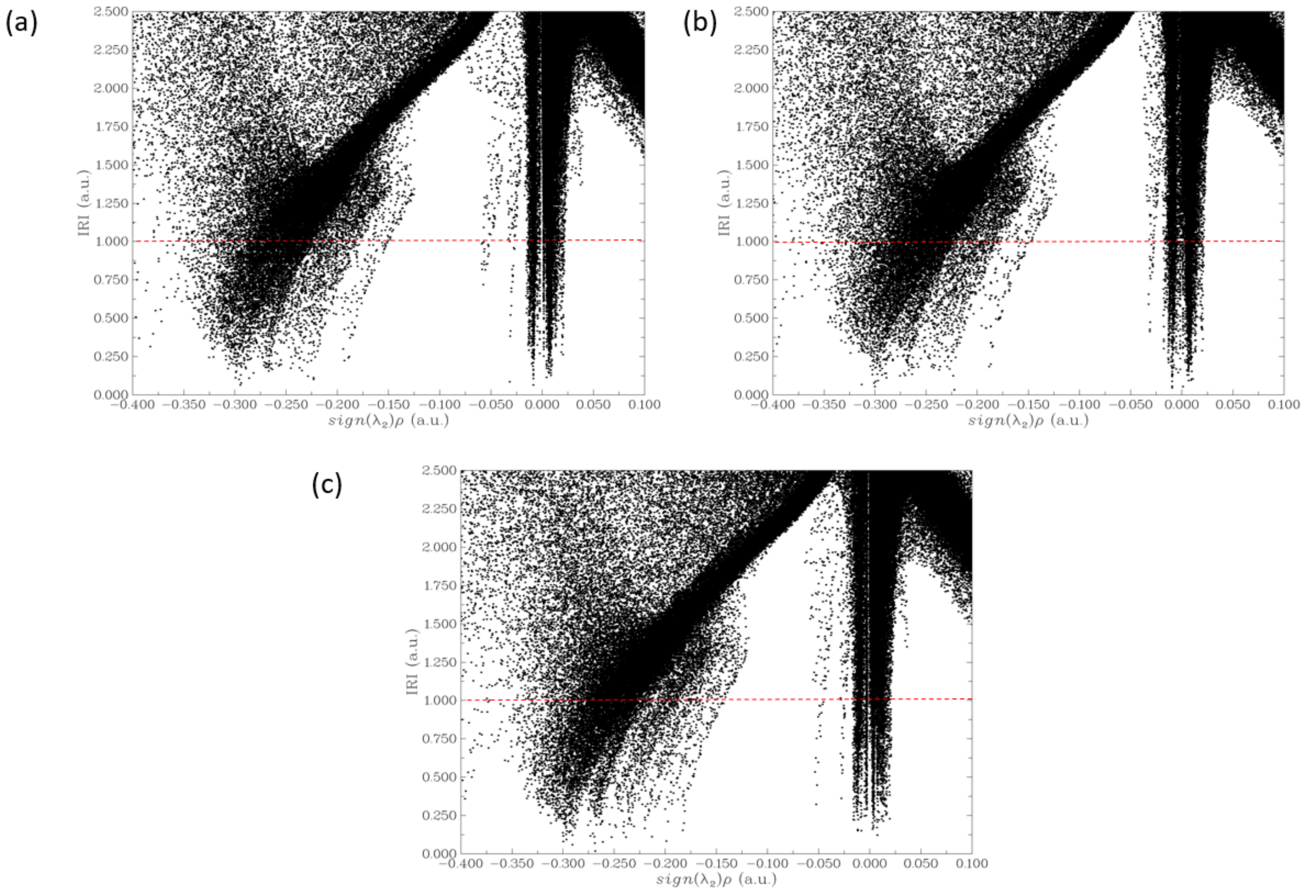

Figure S6. Scatter map of IRI vs sign(λ_2)ρ (-0.4 to 0.1) for *lignin*$^+$ binding with (a) 3-hydroxy-2-naphthoic acid, (b) 6-hydroxy-1-naphthoic acid and (c) 6-hydroxy-2-naphthoic acid. The points intersecting with the red dashed line (IRI = 1.0) correspond to the grid points constituting the isosurfaces shown in **Figure S1**.

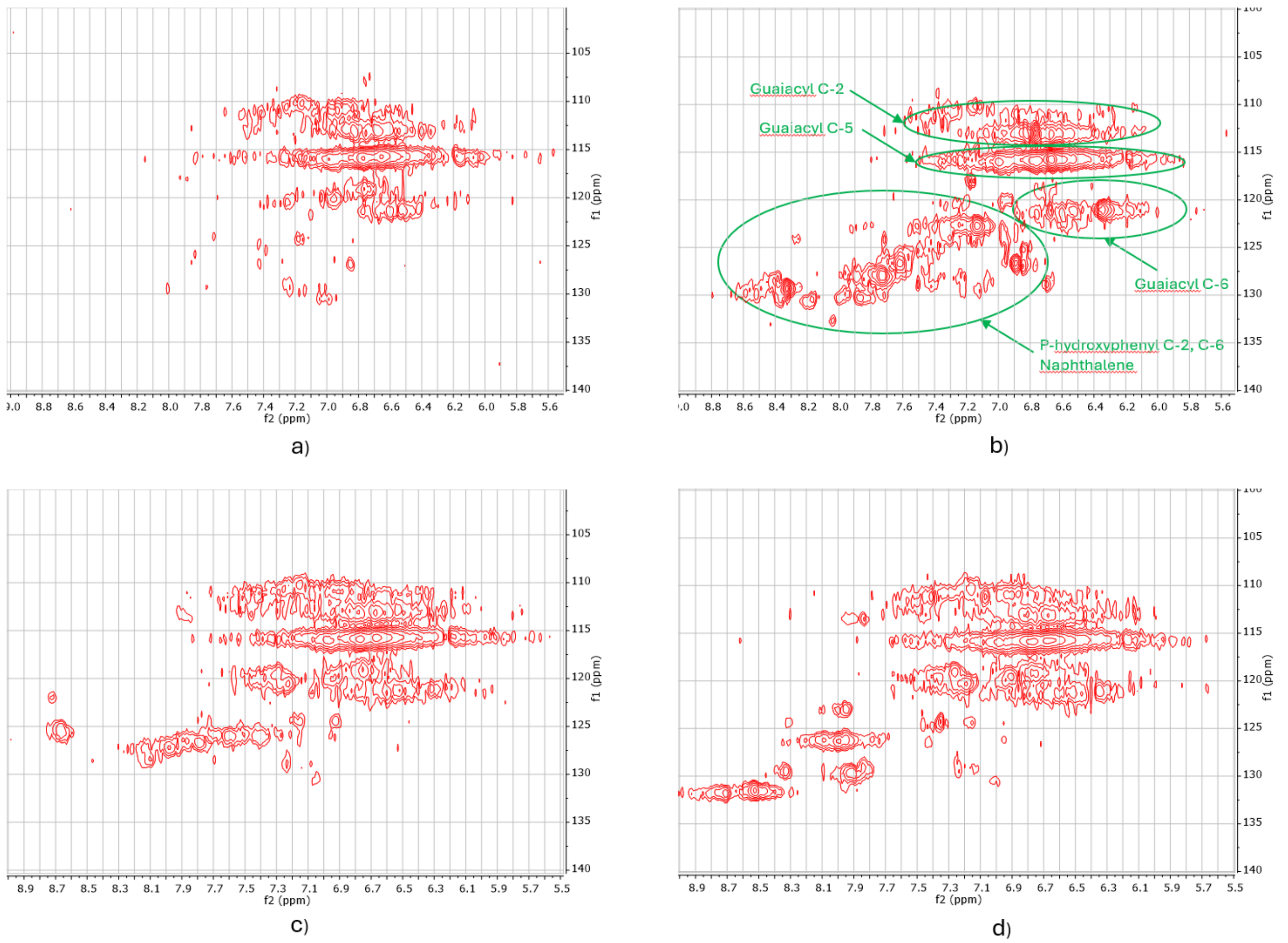

Figure S7. Aromatic region of the ^{13}C–^{1}H HSQC spectra of lignin isolated after pretreatment (a) without additive, (b) with 3-hydroxy-2-naphthoic acid, (c) with 6-hydroxy-1-naphthoic acid and (d) with 6-hydroxy-2-naphthoic acid. Characteristic signals are highlighted exemplarily for the 3-hydroxy-2-naphthoic acid sample.

Table S1. Calculated Gibbs free energy (ΔG) for the binding interaction of $lignin^{+}$, various scavengers and lignin fragment.

Scavengers	Binding Gibbs free energy between $lignin^{+}$ and scavengers or lignin fragment (kJ/mol)
3-Hydroxy-2-naphthoic acid	-16.8
6-Hydroxy-1-naphthoic acid	4.0
6-Hydroxy-2-naphthoic acid	21.7
Lignin fragment	33.3

Table S2. Calculated enthalpy change (ΔH) associated with the binding interaction of $lignin^{+}$ with the investigated cation scavengers and water.

Scavenger	Binding enthalpy between $lignin^{+}$ and scavenger (kJ/mol)	Binding enthalpy between $lignin^{+}$ + scavenger and water (kJ/mol)
3-Hydroxy-2-naphthoic acid	-89.6	-13.4
6-Hydroxy-1-naphthoic acid	-59.3	-13.4
6-Hydroxy-2-naphthoic acid	21.7	-13.7

Table S3. HPLC operating parameters for quantifying hydroxynaphthoic acids (3-hydroxy-2-naphthoic acid, 6-hydroxy-1-naphthoic acid, 6-hydroxy-2-naphthoic acid) and 2-naphthol.

Device / parameter	Detail / value
UHPLC system	Shimadzu Nexera
Column	Nucleodur C8 Gravity, 150×4 mm, 3 µm, 110 Å (Macherey-Nagel #760658.40)
Guard filter	KrudKatcher Ultra in-line filter, 2 µm (Phenomenex #AF0-8497)
Detector (DAD)	Model SPD-M30A, λ=230 nm
Eluent A	Methanol
Eluent B	0.1 % (v/v) H_3PO_4 in H_2O
Gradient	0–10 min: 90% → 40% B 10–16 min: 40% → 20% B 16–19 min: 20% → 90% B
Flow rate	0–13 min: 0.80 mL min^{-1}, > 13 min: 1.20 mL min^{-1}
Column temp.	35 °C
Autosampler temp.	10 °C
Injection volume	10 µL
Calibration range	20–200 mg L^{-1}, quadratic fit through origin, $r^2 > 0.999$
Retention times	3H2NA 13.55 min, 6H1NA 10.60 min, 6H2NA 10.80 min, 2N 12.45 min

Table S4. Compositional analysis of the biomass after pretreatment (210 °C, 2 h) without additive (control) and with carbocation scavengers. Hemicellulose is indicated as mannan. AIL: acid-insoluble lignin, ASL: acid-soluble lignin.

Scavenger	Mannan (%)	Glucan (%)	AIL (%)	ASL (%)
- (control)	0.70% ± 0.07%	49.45% ± 1.13	48.0% ± 1.4%	3.1% ± 0.25%
3-Hydroxy-2-naphthoic acid	0.45% ± 0.05%	37.63% ± 0.26%	58.8% ± 1.4%	3.3% ± 0.07%
6-Hydroxy-1-naphthoic acid	0.84% ± 0.00%	48.67% ± 1.07%	48.8% ± 2.2%	7.62% ± 0.16%
6-Hydroxy-2-naphthoic acid	0.74% ± 0.03%	47.12% ± 0.90%	48.1% ± 1.5%	8.07% ± 0.22%

Table S5. Lignin yields in the extraction of lignin-rich residues with DMSO-d6. Lignin-rich residues were prepared from biomass after pretreatment (210 °C, 2 h) without additive (control) and with carbocation scavengers, by enzymatic cellulose saccharification and ball-milling. Yields refer to the initial lignin content (AIL + ASL) of the residues.

Scavenger	Extraction yield
- (control)	55.3%
3-Hydroxy-2-naphthoic acid	81.6%
6-Hydroxy-1-naphthoic acid	90.4%
6-Hydroxy-2-naphthoic acid	87.6%

Table S6. SEC characterization of the lignins after pretreatment without additive (control) and after pretreatment with carbocation scavengers. Shown are weight average molecular weight (Mw), number average molecular weight (Mn), and polydispersity index (PDI).

Scavenger	Mw	Mn	PDI
- (control)	198259	52176	3.80
3-Hydroxy-2-naphthoic acid	33848	16137	2.10
6-Hydroxy-1-naphthoic acid	25071	12786	1.96
6-Hydroxy-2-naphthoic acid	24067	11589	2.08
